# Regulation of Pannexin 1 Surface Expression by Extracellular ATP: Potential Implications for Nervous System Function in Health and Disease

**DOI:** 10.3389/fncel.2017.00230

**Published:** 2017-08-08

**Authors:** Leigh A. Swayne, Andrew K. J. Boyce

**Affiliations:** ^1^Division of Medical Sciences and Island Medical Program, University of Victoria, Victoria BC, Canada; ^2^Department of Cellular and Physiological Sciences, University of British Columbia, Vancouver BC, Canada

**Keywords:** Pannexin 1, purinergic signaling, P2X7 receptor, ATP, ventricular zone, pain

## Abstract

Pannexin 1 (Panx1) channels are widely recognized for their role in ATP release, and as follows, their function is closely tied to that of ATP-activated P2X7 purinergic receptors (P2X7Rs). Our recent work has shown that extracellular ATP induces clustering of Panx1 with P2X7Rs and their subsequent internalization through a non-canonical cholesterol-dependent mechanism. In other words, we have demonstrated that extracellular ATP levels can regulate the cell surface expression of Panx1. Here we discuss two situations in which we hypothesize that ATP modulation of Panx1 surface expression could be relevant for central nervous system function. The first scenario involves the development of new neurons in the ventricular zone. We propose that ATP-induced Panx1 endocytosis could play an important role in regulating the balance of cell proliferation, survival, and differentiation within this neurogenic niche in the healthy brain. The second scenario relates to the spinal cord, in which we posit that an impairment of ATP-induced Panx1 endocytosis could contribute to pathological neuroplasticity. Together, the discussion of these hypotheses serves to highlight important outstanding questions regarding the interplay between extracellular ATP, Panx1, and P2X7Rs in the nervous system in health and disease.

## Introduction

Recent work from our lab demonstrated that an elevation in extracellular ATP triggers clustering of P2X7Rs and Panx1 leading to endocytosis to intracellular membranes. This regulation of Panx1 surface expression by extracellular ATP has important implications for several physiological and pathophysiological scenarios within the nervous system. Here we present hypotheses describing two scenarios for regulation of cell surface Panx1 expression through putative P2X7R-crosstalk. These include (1) regulation of neural precursor cell (NPC) development within the ventricular zone, and (2) chronic pain and opioid dependence in the spinal cord. First, however, we provide background information on Panx1, extracellular ATP levels, purinergic receptors in the nervous system (primarily P2X7Rs), as well as crosstalk between P2X7Rs and Panx1. Following descriptions of the two proposed scenarios, we conclude with a discussion of knowledge gaps requiring additional insight to better understand the potential for crosstalk between Panx1 and P2X7Rs in the nervous system in health and disease.

### Panx1 and Its Expression in the Nervous System

**FIGURE 1:**
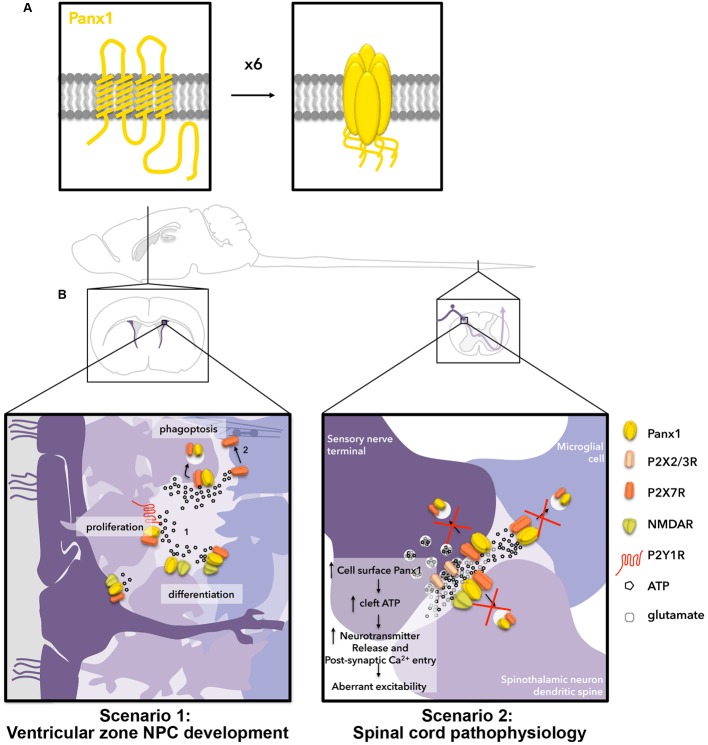
Hypothetical outcomes for two scenarios related to ATP-stimulated internalization of Panx1 channels in the nervous system. **(A)** Panx1 channels (yellow) are formed by hexamers of 4-transmembrane domain Panx1 subunits. **(B)** Two scenarios in which we hypothesize ATP-dependent Panx1 internalization are relevant for nervous system function. Scenario 1 (left) occurs in the postnatal ventricular zone. In the ventricular zone, neural stem cell-like radial glia (dark purple), give rise to rapidly proliferating transit-amplifying NPCs (light purple), which further give rise to neuronal-specific migratory neuroblasts (bluish-purple; left to right). Here, ATP is released by exocytosis and through channels to promote proliferation of transit-amplifying NPCs by activating P2Y1Rs, and to negatively regulate neuronal differentiation/survival by activating P2X7Rs. We have also shown the ATP-release channel Panx1 positively regulates proliferation/maintenance and negatively regulates differentiation (neurite outgrowth) of these cells. NMDARs are also present on radial glia, transit-amplifying NPCs, and neuroblasts, and have been shown to regulate proliferation and differentiation in the ventricular zone. When ATP levels surpass the threshold for internalization, we propose this triggers Panx1 internalization, resulting in reduced proliferation signaling through P2Y1Rs, increased neurite outgrowth (perhaps in part by decreasing P2X7R signaling) and increased phagoptosis by phagocytic neuroblasts. Scenario 2 (right) occurs in the spinal cord. Here, ATP is co-released with glutamate at synapses and is also released from astrocytes (not depicted) and microglia. We propose that ATP-induced Panx1 internalization normally regulates the concentration of ATP in the synapse. We posit that pathological changes to membrane lipid microdomains result in impairment of ATP-mediated endocytosis of Panx1 thereby augmenting Panx1 surface expression and ATP release, resulting in aberrant excitability and leading to the development of chronic pain and opioid withdrawal symptoms.

Panx1 is a four transmembrane domain protein (**Figure 1A**) that was initially discovered (Panchin et al., 2000) through homology to the invertebrate gap junction-forming proteins, innexins. Instead of forming gap junctions, however, Panx1 forms unopposed channels composed of hexamers (reviewed in Sosinsky et al., 2011; Beckmann et al., 2016; Boyce et al., 2017). Panx1 channels mediate ATP release from several different cell types (reviewed in Lohman and Isakson, 2014) and are activated by diverse mechanisms (reviewed in Chiu et al., 2014), such as mechanical stretch (Bao et al., 2004; Xia et al., 2012; Beckel et al., 2014) and caspase cleavage (C-terminus; Sandilos et al., 2012). In the initial investigation of Panx1 distribution, murine Panx1 was most robustly expressed in the CNS (Baranova et al., 2004; Penuela et al., 2007). Panx1 has since been detected in all cell types found in the brain (reviewed in Boyce et al., 2017). Neuronal expression occurs in a wide variety of mature subtypes (Ray et al., 2005; Vogt et al., 2005; Zoidl et al., 2007) and affects physiological and pathophysiological synaptic plasticity (Thompson et al., 2006, 2008; Prochnow et al., 2012; Weilinger et al., 2012, 2016; Ardiles et al., 2014). Panx1 is also expressed in NPCs and immature neurons (Wicki-Stordeur et al., 2012; Wicki-Stordeur and Swayne, 2013), where it is required for NPC maintenance (Wicki-Stordeur et al., 2016) and negative regulation of neurite outgrowth (Wicki-Stordeur et al., 2012, 2016; Wicki-Stordeur and Swayne, 2013; reviewed in Sanchez-Arias et al., 2016). In **Figure 1B** (scenario 1), we depict the potential outcome of ATP regulation of Panx1 surface expression in the context of NPCs in the postnatal ventricular zone. Observations of extra-neuronal (i.e., glial) expression have been more ambiguous. While not originally detected in astrocytes of the healthy mouse (Ray et al., 2005; Vogt et al., 2005; Zappala et al., 2007), a recent study found Panx1 in hippocampal astrocytes (Boassa et al., 2014), supporting its expression in CNS astrocytes. Several reports have investigated the role of Panx1 channels in cultured astrocytes isolated from different areas of the nervous system (reviewed in Freitas-Andrade and Naus, 2016; Boyce et al., 2017), where they have been found to regulate ATP release and participate in neuroinflammatory- (Garré et al., 2010) and pain- (Koyanagi et al., 2016) associated signaling pathways. White matter expression has not yet been resolved (Ray et al., 2005; Weickert et al., 2005), and could possibly reflect axonal transport of transcripts (Sheetz et al., 1998). Panx1 is also found in microglia (Burma et al., 2017) with a recent study revealing its involvement in morphine withdrawal (Burma et al., 2017). In **Figure 1B** (scenario 2), we describe the potential outcome of extracellular ATP regulation of Panx1 surface expression in the context of pain and opioid withdrawal in the dorsal root ganglion and spinal cord.

### Extracellular ATP Levels in the Nervous System

In extracellular spaces within the nervous system, ATP acts as a signaling molecule that can play many different roles. It can act as a fast neurotransmitter, as a trophic factor promoting growth and development, as well as a damage-associated molecular pattern (DAMP; any molecule that can elicit a non-infectious inflammatory response) that regulates communication with phagocytic cells (reviewed in Baroja-Mazo et al., 2013; Chiu et al., 2014; Lohman and Isakson, 2014), including acting as an activator of microglia in the injured cortex (reviewed in Patel et al., 2013). ATP is released (sometimes co-released with GABA and glutamate) into the extracellular space by constitutive and regulated exocytosis from vesicles, through large-pore ion and metabolite channels (Wicki-Stordeur and Swayne, 2012), like Panx1 (reviewed in Dubyak and el-Moatassim, 1993; Abbracchio et al., 2009; Burnstock et al., 2011; Burnstock, 2016b), and from the cytoplasm of damaged/dying cells (reviewed in Wicki-Stordeur and Swayne, 2012). Upregulation of ATP release can occur with increased neuronal activity, with an extreme example being seizure and epilepsy (reviewed in Engel et al., 2016). Synaptic vesicles are predicted to contain a relatively high concentration of ATP (150–200 mM ATP; Van Der Kloot, 2003). Due to the physical constraints imposed by synaptic barriers (Rusakov and Kullmann, 1998), peak ATP concentrations in synaptic clefts following the release of a single ATP-containing vesicle are predicted to reach 500 μM (reviewed in Pankratov et al., 2006). Similar concentrations would be expected in cellular niches in the ventricular zone and spinal cord due to various diffusion barriers. The presence of ectonucleotidases that hydrolyze ATP also restrict ATP levels in a spatial and temporal manner (reviewed in Burnstock, 2016a).

### P2X7Rs Receptors and Their Expression in the Nervous System

Extracellular ATP exerts its effects through concentration-dependent activation of various combinations of ionotropic P2XRs and metabotropic P2Y receptors (P2YRs, reviewed in Burnstock, 2011; Cavaliere et al., 2015). P2XRs (P2X1R-P2X7R) are cation-permeable channels (Ca^2+^, Na^+^, K^+^) formed from trimers of individual subunits that consist of intracellular N- and C-termini, two transmembrane domains, and a large, highly conserved extracellular domain that contributes to the intersubunit ATP-binding pocket (Ennion et al., 2000; Jiang et al., 2000; Wilkinson et al., 2006; Yan et al., 2006; Fischer et al., 2007; Zemkova et al., 2007; Roberts et al., 2008; Evans, 2009; Kawate et al., 2009; Browne et al., 2010; Hattori and Gouaux, 2012; Chataigneau et al., 2013). ATP binding to this pocket causes a conformational change that leads to pore opening. A growing body of research has revealed particularly strong links between Panx1 and P2X7Rs (reviewed in Isakson and Thompson, 2014; Bravo et al., 2015).

Within the CNS, P2X7R expression has been detected at the transcript and protein levels in neurons, astrocytes, and microglia across the brain and spinal cord (reviewed in Cotrina and Nedergaard, 2009), suggesting P2X7R expression overlaps with Panx1, at least partially, although this remains to be confirmed as several issues with antibodies and knockout mice have made establishing the definitive expression and function of P2X7Rs in neuronal subtypes challenging (discussed in Metzger et al., 2016). A recent study created a humanized conditional mouse to genetically dissect P2X7R expression within the central nervous system (Metzger et al., 2016). The results of this study suggested that neuronal P2X7Rs could be specific to glutamatergic neurons of the CA3 region of the hippocampus, and at very low levels in cortex and cerebellum, present mainly in non-neuronal cells (astrocytes, oligodendrocytes and microglia). Interestingly, however, the strong expression at the mRNA level in the CA3 was not observed in a different reporter mouse line (García-Huerta et al., 2012; Hirayama et al., 2015; Jimenez-Mateos et al., 2015). Thus, the precise localization of P2X7Rs within neurons in the CNS might still be considered somewhat controversial.

P2X7Rs are also present in NPCs and NPC model cell lines, where they play key roles in maintenance of stemness, proliferation, differentiation and programmed cell death (reviewed in Burnstock and Ulrich, 2011; Cavaliere et al., 2015). In N2a cells, a model of neuronal differentiation that we used to study Panx1 trafficking in (Boyce et al., 2015; Boyce and Swayne, 2017), P2X7Rs are the primary functional P2XR subtype (Gomez-Villafuertes et al., 2009). P2X7Rs are expressed in the embryonic (Tsao et al., 2013) and postnatal (Messemer et al., 2013) ventricular zone, as well as the early postnatal subgranular zone (Tsao et al., 2013), an NPC niche within the hippocampus. In model NPC cell lines, a decrease in P2X7R expression was associated with neuronal commitment (Wu et al., 2009; Orellano et al., 2010; Glaser et al., 2014) suggesting negative regulation. As follows, receptor antagonism and knock-down induced neurite outgrowth (Gomez-Villafuertes et al., 2009; Wu et al., 2009) and branching (Díaz-Hernandez et al., 2008). In contrast, P2X7Rs promoted differentiation in the embryonic ventricular zone (Tsao et al., 2013), while in the postnatal ventricular zone (Messemer et al., 2013), P2X7Rs have also been shown to promote cell death to limit the possibility of over-proliferation. Similarly, P2X7Rs promoted death during differentiation conditions in human SH-5YSY neuroblastoma cells (Orellano et al., 2010). Conversely, in N2a cells, P2X7Rs promoted survival during serum- and glucose-deprived conditions (Gómez-Villafuertes et al., 2015). In addition to promoting cell death, another manner in which P2X7Rs have been proposed to regulate NPC populations is through phagoptosis, cell death through phagocytosis by neighboring phagocytic NPCs (Lu et al., 2011; Brown and Neher, 2012). NPC phagoptosis has been shown to occur through a non-canonical P2X7R-dependant mechanism, involving an interaction with myosin that is inhibited by extracellular ATP (Lovelace et al., 2015). Together these finding suggest that P2X7Rs regulate neuronal differentiation and survival in a developmentally regulated manner. The differential effects of P2X7Rs across these NPC contexts suggests that there could be other factors involved, such as differences in extracellular ATP levels or other proteins (i.e., Panx1) involved in their crosstalk that are developmental stage- and/or model-specific (see Gampe et al., 2015; Kaebisch et al., 2015). These concepts will be revisited later in scenario 1, “Implications of ATP-induced Panx1 internalization in ventricular zone NPCs.” It should be noted that P2YRs also play an important role in regulating NPC behavior. P2YRs are G-protein coupled receptors (GPCRs) that respond primarily to ADP, UTP, and UDP, with lower affinity for ATP (reviewed in Weisman et al., 2012). P2YRs couple to G_q_, G_s_ or G_i_ (reviewed in Erb et al., 2006) to modulate intracellular Ca^2+^ and cAMP. Suyama et al. (2012) found that P2Y1R regulated the proliferation of rapidly dividing (“transit-amplifying”) NPC subtypes within the adult mouse ventricular zone. Other examples include P2Y2R (Arthur et al., 2005, 2006) and P2Y4R (Cavaliere et al., 2005), which have been associated with neuronal differentiation.

### P2X7R-Panx1 Crosstalk, Including ATP-Mediated Panx1 Endocytosis

Crosstalk between P2X7Rs and Panx1 occurs in several cell types (reviewed in Isakson and Thompson, 2014; Bravo et al., 2015) within diverse physiological and pathophysiological contexts (reviewed in Baroja-Mazo et al., 2013). It should be noted here that the relationship between the P2X7R pore and Panx1 is somewhat controversial: some studies attribute the large pore formed by P2X7R to Panx1, while other studies refute this (reviewed in Baroja-Mazo et al., 2013). It is generally accepted that P2X7R activation (increasing intracellular Ca^2+^ (Garre et al., 2016) or activating Src kinase; Iglesias et al., 2008) can enhance Panx1 function and thus crosstalk between Panx1 and P2X7Rs occurs within the context of a positive feedback loop. Examples of this include regulation of neuronal activity in the supraoptic nucleus (Ohbuchi et al., 2011), enteric neuronal death (Gulbransen et al., 2012), and neuroinflammasome activation (Silverman et al., 2009). A number of studies have observed a physical interaction between P2X7Rs and Panx1 (Pelegrin et al., 2006; Silverman et al., 2009; Poornima et al., 2012; Hung et al., 2013; Kanjanamekanant et al., 2014; Pan et al., 2015; Seref-Ferlengez et al., 2016). Initially, their interaction was observed within the inflammasome complex (Silverman et al., 2009). Mechanical stress also induced their interaction (Kanjanamekanant et al., 2014). Notably, there are multiple P2X7R splice variants and single nucleotide polymorphisms in both human (Cheewatrakoolpong et al., 2005; Adinolfi et al., 2010) and mouse (Masin et al., 2012; Kido et al., 2014) genes (reviewed in Costa-Junior et al., 2011; Sperlagh and Illes, 2014). Several studies have shown that expression of these variants can modulate functional crosstalk with Panx1 (Adinolfi et al., 2010; Masin et al., 2012). However, it is currently unknown whether the specific P2X7R isoform affects physical coupling between Panx1 and P2X7R; determination of the site of interaction on the P2X7R could help bridge this gap in knowledge.

Adding further complexity to P2X7R-Panx1 crosstalk, we recently demonstrated that elevation of extracellular ATP leads to Panx1 internalization (Boyce et al., 2015; Boyce and Swayne, 2017), thereby reducing Panx1 surface expression. ATP-induced internalization required activation of P2X7Rs (Boyce et al., 2015) as well as their physical interaction with the Panx1 first extracellular loop (Boyce and Swayne, 2017). This was the first report to identify the interaction site for the P2X7R within the Panx1 sequence. Although P2X7R activation was required, thorough analysis of intracellular P2X7R-dependent intracellular signaling pathways (Src and Ca^2+^) revealed that these played no role in ATP-induced P2X7R-Panx1 clustering and internalization. Importantly, removal of extracellular ATP with apyrase (to hydrolyze endogenously released ATP) completely abolished Panx1-P2X7R clustering. Cholesterol-disrupting agents blocked clustering and endocytosis, and endocytosis was dynamin-independent, suggesting a clathrin-independent mechanism. While the physiological implications of ATP-induced internalization are currently under investigation in the lab, here we describe two scenarios where it is likely to occur: within the NPC populations in the ventricular zone, as well as within the spinal cord (and dorsal root ganglion) in the context of neuropathic pain and morphine withdrawal.

## Scenario 1: Atp-Mediated Panx1 Endocytosis in the Regulation of Cellular Behaviors in the Adult Ventricular Zone

Neural precursor cells in the adult ventricular zone consist of three different developmental stages (reviewed in Lim and Alvarez-Buylla, 2016). **Figure 1B**, scenario 1 depicts these cells. The slowly dividing “radial-glia”-like NPCs (dark purple) line or extend processes to the ventricular surface along with ependymal cells. These give rise to rapidly dividing “transit-amplifying” NPCs (further right, lighter purple), and neuronally committed, doublecortin (DCX)-positive neuroblasts (right-most, bluish-purple). Panx1 and P2X7Rs can be found in each of these cell types. Notably, *N*-methyl-D-aspartate receptors (NMDARs) can also be found across the developmental cell types (reviewed in Jansson and Akerman, 2014). Furthermore, ATP is episodically released from both NPCs and astrocytes (not depicted) within this niche (Lacar et al., 2012; Suyama et al., 2012), making this a relevant system for ATP-dependent Panx1 internalization (Khodosevich et al., 2012; Suyama et al., 2012). While the source of this ATP has not yet been comprehensively defined, our work suggested it could at least in part derive from Panx1-mediated release (Wicki-Stordeur et al., 2012, 2016; Wicki-Stordeur and Swayne, 2013; reviewed in Swayne and Bennett, 2016). We propose that ATP-evoked Panx1 internalization is a mechanism to keep ATP-dependent processes in check. Once extracellular ATP levels reach a certain upper threshold (∼200 μM), our recent findings predict that Panx1 internalizes (following ATP-induced interaction with P2X7Rs) on nearby NPCs (**Figure 1B**) to prevent further ATP release. There are several potential consequences of ATP-induced internalization with the ventricular zone; these are depicted in **Figure 1B**, scenario 1.

### NPC Proliferation and Differentiation (**Figure 1B**, Scenario 1, Part 1)

As described above, one major role identified for extracellular ATP, is to promote the proliferation of transit-amplifying NPCs through the activation of P2Y1Rs (Suyama et al., 2012). The proliferation of these cells is coupled to local increases in blood flow (Lacar et al., 2012). P2Y1Rs are coupled to G_q_ and thus when activated lead to IP_3_ receptor-dependent increases in intracellular Ca^2+^. Increased intracellular Ca^2+^, in turn, augments Panx1-mediated ATP release (Locovei et al., 2006). Since the impact of ATP on NPC proliferation creates this potential positive feedback loop (that conceivably leads to tumor formation), it would be reasonable to speculate that ATP-induced P2X7R-Panx1 clustering and endocytosis helps prevent over-proliferation, by reducing extracellular ATP. ATP-induced P2X7R-Panx1 clustering and endocytosis could also impact on NPC differentiation through regulating surface expression of Panx1 and P2X7Rs, which negatively regulate differentiation. We recently showed that blocking or knocking down Panx1 induces robust neurite outgrowth and stabilization in NPCs (Wicki-Stordeur and Swayne, 2013); we now need to investigate whether reduction of surface expression also induces neurite outgrowth (whether through disrupting cell surface signaling or through modifying the function of endosomes, as described below). Membrane trafficking is a critical component of neurite outgrowth, which is negatively regulated by Panx1 (Wicki-Stordeur and Swayne, 2013). Endocytosis of signaling molecules such as growth factor receptors, regulates where and when signaling cascades are triggered (reviewed in Yap and Winckler, 2012). In addition to regulating extracellular ATP concentrations, Panx1 endocytosis likely also regulates intracellular Panx1 signaling. Although Panx1-associated intracellular signaling cascades are still relatively poorly characterized, this could, for example, include crosstalk with the actin cytoskeleton (Bhalla-Gehi et al., 2010; Wicki-Stordeur and Swayne, 2013), or function of recycling endosomes where internalized Panx1 resides in the short term (Boyce et al., 2015). At the recycling endosome, Panx1 could couple to proteins restricted to the endosomal lumen (via the Panx1 extracellular loops) or to proteins tethered to the cytoplasmic leaflet of the endosomal compartment, to regulate processes like membrane trafficking. Moreover, Panx1 also exhibits physical and functional crosstalk with NMDARs (Weilinger et al., 2012, 2016), the activation of which increases proliferation and differentiation of NPCs of a variety of origins (Deisseroth et al., 2004; Joo et al., 2007; Yoneyama et al., 2008; Cho et al., 2013) including postnatal ventricular zone NPCs (Fan et al., 2012). NMDAR activation by local astrocytic glutamate release is also critical for neuroblast survival (Platel et al., 2010). The putative role of signaling interplay between these three physically and functionally linked proteins (P2X7R, NMDAR, Panx1) in the context of the ventricular zone will shed important light on the regulation of NPC proliferation and differentiation.

### NPC Clearance (**Figure 1B**, Scenario 1, Part 2)

We recently demonstrated that selective deletion of Panx1 in ventricular zone NPCs led to their loss over time (Wicki-Stordeur et al., 2016). We proposed that Panx1 is needed for release of ATP, which acts as a “don’t-eat-me” signal warding off neighboring phagocytic DCX-positive neuroblasts (Lovelace et al., 2015). Here, a non-canonical P2X7R-dependent signaling pathway (Gu et al., 2010, 2011) involving a physical interaction between P2X7Rs and non-muscle myosin (purple barbell interposed between actin filaments) regulates neuroblast-mediated phagocytosis (Lovelace et al., 2015), also referred as phagoptosis (Lu et al., 2011). It should be noted that contrary to what might be expected microglia do not phagocytose NPCs within the ventricular zone but instead support their survival (Ribeiro Xavier et al., 2015). Extracellular ATP inhibits the interaction between P2X7Rs and non-muscle myosin (Gu et al., 2010) within phagocytic neuroblasts, thereby inhibiting neuroblast-mediated phagoptosis (Lovelace et al., 2015). Thus a rise in ATP above a certain threshold would trigger removal of surface Panx1 resulting in a decrease in extracellular ATP. This decreases proliferative signaling through P2Y1R and also potentially renders these NPCs susceptible to phagoptosis, immediately keeping the size of the transit-amplifying NPC population in check. In the context of cortical injury (not depicted in **Figure 1B**, scenario 1), NPCs migrate to the injured cortex, a totally different cellular environment where microglia (not neuroblasts) are now the phagocytic cells. Here, ATP acts as a DAMP/“find-me-eat-me” signal, activating microglia through metabotropic P2Y12Rs (Haynes et al., 2006). Thus, here we expect Panx1 expression to be deleterious, as supported by our recent study, where deletion of Panx1 improved NPC survival in the peri-infarct cortex (Wicki-Stordeur et al., 2016).

## Scenario 2: Disruption Of Atp-Mediated Panx1 Endocytosis in Chronic Pain and Morphine Tolerance in the Spinal Cord

“Normal” nociception, also commonly known as pain sensation, is a distressing feeling caused by an intense or damaging stimulus that normally resolves when the stimulus is removed. Chronic pain, on the other hand, is pathologically persistent and can include hypersensitivity, a pain sensation that is greater than would be expected with a given stimulus, as well as allodynia, a sensation of pain caused by a non-painful stimulus. Chronic pain arises from an ongoing inflammatory response and is associated with complex functional remodeling within sensory circuits. Chronic pain is often associated with neuropathic pain (pain arising from injury to the nerves themselves). Within the spinal cord, P2X7R activity has been reported, both pre- and post-synaptically, to impact on neurotransmitter release and synaptic currents (**Figure 1B**, scenario 2, reviewed in Cotrina and Nedergaard, 2009). The subcellular localization of Panx1 within the different cell types of the spinal cord is not currently known. Speculation of the involvement of Panx1 in chronic pain (Bravo et al., 2014, 2015) originated from the previous understanding of ATP and P2X7Rs as established molecular regulators of spinal cord injury (Wang et al., 2004) and associated chronic pain (Chessell et al., 2005; Honore et al., 2006; McGaraughty et al., 2007; reviewed in Tsuda and Inoue, 2016). Several studies have approached the investigation of the putative role of Panx1 in chronic pain from different angles and with varying results.

An early study in rats by Bravo et al. (2014) found no evidence for a role of Panx1 in normal nociception; however, Panx1 blockers decreased “wind-up” (an electrophysiological phenomenon associated with the development of chronic pain) in a spared nerve injury model of neuropathic pain (axotomy of 2 of 3 sciatic nerve terminal branches). While these authors found no change in Panx1 expression in the spinal cord proper associated with their neuropathic pain model, a subsequent study by Zhang et al. (2015) using a rat sciatic spinal nerve ligation model found increased expression of Panx1 in NeuN-positive DRG neurons associated with Panx1 promoter modulation. These authors similarly described a reduction in pain hypersensitivity associated with disrupting Panx1 (block and siRNA). Another study (Koyanagi et al., 2016) identified Panx1 as the source of ATP released in the spinal cord in the context of glucocorticoid-mediated diurnal enhancement of pain sensitivity. These authors used a mouse partial sciatic nerve ligation hypersensitivity model. Here, Panx1-mediated ATP release was attributed to spinal cord astrocytes. Finally, the most recent work in this area dissected the cell-type specific role of Panx1 in sciatic nerve-injury associated neuropathic pain at the cellular level using a number of Cre-lines. After confirming that global Panx1 knockout mice are protected from the development of neuropathic pain (Weaver et al., 2017), they next ruled out the contribution neuronal and astrocytic Panx1 to the development of sciatic nerve-injury based hypersensitivity using Syn-Cre (targeting neurons) and GFAP-Cre (targeting astrocytes) lines crossed with floxed Panx1 mice. Further, since Panx1 is also expressed in immune cells that are upregulated in the spinal cord in the context of neuropathic pain, they performed bone marrow transplantation studies to test the hypothesis that bone-marrow derived immune cell-Panx1 contributes to the development of neuropathic pain. Remarkably, when Panx1 wildtype bone marrow was transplanted into Panx1 knockout mice subjected to spared nerve injury, hypersensitivity was restored, indicating that bone-marrow derived immune cells were indeed the source of the Panx1 associated with the development of neuropathic pain. Subsequent analyses surprisingly argued against macrophage or microglial contributions, implying either compensation or involvement of another bone marrow-derived cell type. Together, these studies strongly implicated Panx1-mediated ATP release within the spinal cord (or nearby dorsal root ganglion) as a key element of the development and/or modulation of chronic pain; however, the specific cell type(s) involved have yet to be fully elucidated. Relatedly, opioid withdrawal was recently shown to be mediated by Panx1 (Burma et al., 2017). In this study, genetic dissection attributed withdrawal to dorsal horn microglia, where Panx1 (and P2X7R) levels were increased and Panx1-mediated ATP release resulted in the development of morphine withdrawal. While changes in overall Panx1 expression levels were equivocal amongst these studies, in light of our recent findings, potential alterations in Panx1 surface expression should also be investigated.

In the context of the healthy spinal cord, we hypothesize that, like with NPCs and proliferation, ATP-mediated P2X7R-Panx1 clustering and internalization normally acts as a safeguard in the context of pain signaling. ATP-mediated Panx1 internalization relies on activation of and interaction with P2X7Rs, and both are present on spinal cord microglia, astrocytes, and neurons. Therefore all of these cell types are potential loci where ATP-induced Panx1 internalization occurs. We hypothesize that this ATP-mediated regulation of Panx1 is disrupted by molecular and cellular changes associated with the development of neuropathic pain and/or morphine tolerance (**Figure 1B**, scenario 2). The outcome of impaired Panx1 internalization would be increased surface expression and activity; this could also potentially contribute to the observed upregulation of Panx1 (albeit that data was equivocal) if delaying internalization also delays degradation (although this has yet to be fully investigated).

ATP-induced P2X7R-Panx1 clustering and internalization was robustly inhibited by cholesterol-disrupting agents, suggesting a requirement of specialized cholesterol-rich lipid membrane microdomains (also known as lipid rafts) for ATP-induced internalization (currently under investigation in our lab). Several recent studies have shown that lipids enriched in these membrane microdomains are disrupted in neuropathic pain. For example, the expression of a key enzyme in cholesterol synthesis hydroxymethylglutaryl-CoA synthase 1 (HMGCS1), is downregulated within the DRG after spinal nerve injury (Wang et al., 2016). Another study (Patti et al., 2012) also identified the dysregulation of sphingolipids, another class of lipids co-enriched in cholesterol-rich membrane microdomains (reviewed in Bou Khalil et al., 2010) in the ipsilateral dorsal horn during chronic neuropathic pain. In the context of morphine withdrawal, membrane cholesterol is also a key regulator of opioid receptor signaling (Zheng et al., 2012). If opioid exposure in turn modulates membrane cholesterol, this would disrupt ATP-induced Panx1 internalization in multiple cell types (including those not depicted, such as astrocytes), potentially accounting for the observed abnormal Panx1 levels and Panx1-mediated ATP release (Burma et al., 2017). It is reasonable to speculate that this reduction in Panx1 endocytosis in cells within the spinal cord would, over time, increase relative Panx1 surface expression and ATP release. Increased synaptic ATP would, in turn, act through presynaptic P2XRs, namely P2X2R and P2X3R, to increase neurotransmitter release at dorsal horn synapses (Li and Perl, 1995; Gu and MacDermott, 1997; Li et al., 1998; Nakatsuka and Gu, 2001; Nakatsuka et al., 2003), and also increase Ca^2+^ entry through P2XRs postsynaptically, as depicted in **Figure 1B**, scenario 2. In summary, while the impact of these lipid changes on ATP-induced internalization needs to be confirmed experimentally, we hypothesize that such lipid changes impair ATP-induced internalization, thereby contributing to the development of neuropathic pain and potentially also morphine tolerance (**Figure 1B**, scenario 2).

## Conclusion

Our recent work has shown that elevation of extracellular ATP triggers clustering of Panx1 with P2X7Rs and subsequent internalization (Boyce et al., 2015; Boyce and Swayne, 2017). In this hypothesis paper, we have outlined two scenarios where we predict this down-regulation of Panx1 surface expression could play an important role. The first scenario pertained to the regulation of NPC development and maintenance within the ventricular zone, where downregulation of Panx1 at the cell surface could lead to decreased proliferation, and increased differentiation and/or neurite outgrowth, as well as increased susceptibility to phagoptosis. A more detailed understanding of the crosstalk between Panx1, P2X7R, and NMDAR over the course of neuronal development within the ventricular zone will require more precise knowledge of their cell-type specific relative expression levels as well as the molecular determinants of the interactions between these transmembrane proteins and additional signaling proteins. The second scenario involved chronic pain and opioid dependence in the spinal cord, where we predict ATP-induced endocytosis of Panx1 is impaired, possibly due to pathogenic changes in membrane lipids, leading to the observed upregulation in Panx1-mediated ATP release. Analysis of Panx1 surface expression within the context of chronic pain and/or opiate withdrawal and characterization of cell-type specific changes in lipid profiles are now required to investigate this hypothesis. Overall, the discovery of ATP-induced internalization of Panx1 provides new understanding of regulation of extracellular ATP, Panx1, and crosstalk between Panx1 and P2X7R, with broad implications for nervous system function.

## Author Contributions

LS and AB conceived of the initial topic. LS wrote the manuscript and created the figure. AB assisted with writing and figure creation.

## Conflict of Interest Statement

The authors declare that the research was conducted in the absence of any commercial or financial relationships that could be construed as a potential conflict of interest.
